# High early mortality following percutaneous nephrostomy in metastatic cancer: a national analysis of outcomes

**DOI:** 10.1136/spcare-2024-004937

**Published:** 2024-07-13

**Authors:** Amandeep Dosanjh, Benjamin Coupland, Jemma Mytton, Dominic Stephen King, Harriet Mintz, Anna Lock, Veronica Nanton, Param Mariappan, Nigel Trudgill, Prashant Patel

**Affiliations:** 1 University of Birmingham Institute of Cancer and Genomic Sciences, Birmingham, UK; 2 Research and Development, University Hospitals Birmingham NHS Foundation Trust, Birmingham, UK; 3 Department of Gastroenterology, The Dudley Group NHS Foundation Trust, Dudley, West Midlands, UK; 4 School of Medical and Dental Sciences, University of Birmingham, Birmingham, UK; 5 Department of Palliative Care, Sandwell and West Birmingham Hospitals NHS Trust, Birmingham, UK; 6 Department of Social Sciences and Systems in Health, University of Warwick, Coventry, UK; 7 Edinburgh Bladder Cancer Surgery (EBCS), Department of Urology, Western General Hospital, Edinburgh, UK; 8 Department of Gastroenterology, Sandwell and West Birmingham Hospitals NHS Trust, Birmingham, UK; 9 Department of Urology, University College London Hospitals NHS Foundation Trust, London, UK

**Keywords:** Cancer, Genitourinary, Clinical decisions, End of life care, Palliative Care, Quality of life

## Abstract

**Objectives:**

To assess the outcomes of percutaneous nephrostomy in England for renal decompression, in the context of metastatic cancer.

**Methods:**

Retrospective observational study of all patients undergoing nephrostomy with a diagnosis of metastatic cancer from 2010 to 2019 in England, identified and followed up within Hospital Episode Statistics.

The primary outcome measure was mortality (14-day and 30-day postprocedure). Secondary outcomes included subsequent chemotherapy or surgery and direct complications of nephrostomy.

**Results:**

10 932 patients were identified: 58.0% were male, 51.0% were >70 years old and 57.7% had no relevant comorbidities (according to Charlson’s criteria, other than cancer).

1 in 15 patients died within 14 days of nephrostomy and 1 in 6 died within 30 days. Factors associated with higher 30-day mortality were the presence of comorbidities (Charlson score 1–4 (OR 1.27, 95% CI 1.08 to 1.50, p=0.003), score 5+ (OR 1.29, 95% CI 1.14 to 1.45), p<0.001)); inpatient nephrostomy (OR 3.76, 95% CI 2.75 to 5.14, p<0.001) and admitted under the care of specialities of internal medicine (OR 2.10, 95% CI 1.84 to 2.40, p<0.001), oncology (OR 1.80, 95% CI 1.51 to 2.15, p<0.001), gynaecology/gynaeoncology (OR 1.66, 95% CI 1.21 to 2.28, p=0.002) or general surgery (OR 1.62, 95% CI 1.32 to 1.98, p<0.001)), compared with urology.

25.4% received subsequent chemotherapy. Receiving chemotherapy was associated with younger patients (eg, age 18–29 (OR 4.04, 95% CI 2.66 to 6.12, p<0.001) and age 30–39 (OR 3.07, 95% CI 2.37 to 3.97, p<0.001)) and under the care of oncology (OR 1.60, 95% CI 1.40 to 1.83, p<0.001) or gynaecology/gynaeoncology (OR 1.64, 95%CI 1.28 to 2.10, p<0.001) compared with urology.

43.8% had subsequent abdominopelvic surgery. Not receiving surgery was associated with inpatient nephrostomy (OR 0.82, 95%CI 0.72 to 0.95,p=0.007): non-genitourinary cancers (eg, gynaecology/gynaeoncology cancer (OR 0.86, 95% CI 0.74 to 0.99, p=0.037)); and under the care of a non-surgical specialty (medicine (OR 0.69, 95% CI 0.63 to 0.77, p<0.001), oncology (OR 0.58, 95% CI 0.51 to 0.66, p<0.001)).

24.5% of patients had at least one direct complication of nephrostomy: 12.5% required early exchange of nephrostomy, 8.1% had bleeding and 6.7% had pyelonephritis.

**Conclusions:**

The decision to undertake nephrostomy in patients with poor prognosis cancer is complex and should be undertaken in a multidisciplinary team setting. Complication rates are high and minimal survival benefit is derived in many patients, especially in the context of emergency inpatient care.

**WHAT IS ALREADY KNOWN ON THIS TOPIC**

- Malignant ureteric obstruction (MUO) in the context of metastatic cancer indicates a poor prognosis.
- Nephrostomy is commonly used for renal decompression in these cases but rarely confers survival benefit.
- Living with a nephrostomy is associated with complications and a reduced quality of life.

**WHAT THIS STUDY ADDS**

- This study provides a large cohort at a national level, considering the outcomes for patients with MUO in the context of metastatic disease.
- As this is a Hospital Episode Statistics-based study, it provides a review of national practice and factors associated with poor prognosis.
- The extremely poor mortality, especially in the context of emergency nephrostomy, displayed in this study will aid clinicians in being more selective when offering patients nephrostomy when presenting with MUO.
- This study has displayed that few patients go on to have further treatment, therefore, significant numbers of patients receive a quality-of-life altering nephrostomy with no benefit in the form of treatment or survival.

**HOW THIS STUDY MIGHT AFFECT RESEARCH, PRACTICE OR POLICY**

- Future research may be able to identify those indications where nephrostomy may offer survival benefit.
- Patients managed by urology or oncology teams, as a primary speciality, at the time of nephrostomy insertion have better-predicted outcomes. Therefore, this research will empower clinical teams to seek early involvement urology and oncology teams for decision-making, in order to improve patient outcomes.
- This study gives insight into the national burden of renal decompression in the context of metastatic cancer. It can help direct resource allocation and national policy with regard to this patient population.

## Introduction

 Percutaneous nephrostomy is routinely used to decompress obstructed renal systems; along with ureteric stenting. It allows the drainage of urine directly from the renal pelvicalyceal region, thus diverting urine away from more distal obstruction.

Malignant ureteric obstruction (MUO) is a clinical indication of advanced or disseminated cancers. The benefit of renal decompression in patients with a limited prognosis is contentious, with no clear guidance available for such situations.^1^ While there may be some advantage to avoiding the sequelae of postrenal obstruction, such as uraemia, electrolyte imbalance or infection, it is unclear what benefit many of these patients derive from nephrostomy in end-of-life care settings. Survival benefit has only been demonstrated in specific malignancies and only for those with slow-growing cancers.^2^

The aim of this study was to examine the outcomes including short-term survival and complications of nephrostomy in England for patients with malignant disease. Associations with short-term mortality, subsequent surgery and chemotherapy were examined.

## Methods

### Data source

Hospital Episode Statistics (HES) is a nationally curated administrative database of all patients interacting with publicly funded hospital care in England. Data are organised longitudinally by hospital episode; the period of time under the care of a single consultant team. In addition to demographic details, information pertaining to operative procedures and diagnostic records is stored as Office of Population Census and Surveys Classification of Interventions and Procedures, version 4 codes, and International Classification of Diseases version 10 codes, respectively.

### Inclusion criteria

All patients undergoing nephrostomy with a diagnosis of metastatic cancer, diagnosed at any time prior to nephrostomy or within 3 months following nephrostomy, with the presence of a metastatic cancer code, between January 2010 and December 2019.

### Exclusion criteria

**Figure 1:**
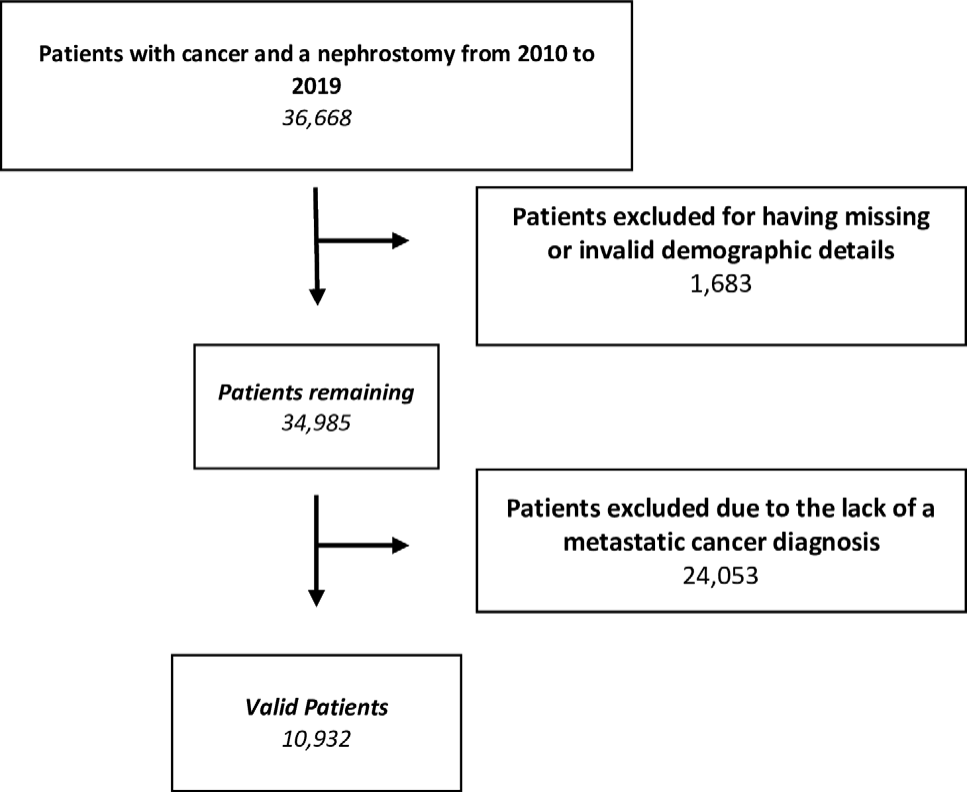
Study flow chart.

Patients were excluded for age <18, living outside of England and for missing data and follow-up. Further exclusions were made for lack of a metastatic cancer diagnosis (figure 1).

### Demographic data

Age, sex, residential region, Index of Multiple Deprivations (IMD) 2010 quintile and ethnicity were included as demographic data. Charlson Comorbidity Score was calculated, with a modification to exclude a diagnosis of cancer; this has been validated in previous HES-based studies.^3 4^

### Outcome measures

The primary outcome measure was 30-day mortality following index nephrostomy insertion. Secondary outcomes were receiving chemotherapy or further abdominopelvic surgery following nephrostomy and direct complications of nephrostomy.

Complications of nephrostomy were identified as pyelonephritis, bleeding/haematuria or early (within 2 months) exchange/resiting of nephrostomy.

Outpatient nephrostomy was defined as those patients attending electively as an outpatient or day case; with less than 1 night stay in hospital. Emergency admissions are flagged within the HES dataset.

Provider volume was identified as the total volume of cancer patients treated at the respective provider over the study period and was split into tertiles: low (1–1576 patients), medium (1577–3079) and high volume (3080+).

Site of cancer was split by primary tumour: genitourinary; gynaecological oncology; lower-gastrointestinal and all others. The primary specialty assigned to the index nephrostomy episode was considered to be the nephrostomy organising specialty.

### Statistical analysis

Multivariable logistic regression models were constructed in order to consider the association of demographic and clinically relevant variables. Outcomes were likelihood of 30-day mortality, receiving further chemotherapy and receiving further abdominopelvic surgery. Codes for percutaneous procedures, purely diagnostic procedures and minor procedures not requiring regional or general anaesthetic were excluded.

An unadjusted Kaplan-Meier curve following survival postnephrostomy for 6 months was constructed.

CIs were set at 95% and a p<0.05 was considered statistically significant. All data extraction was performed in Microsoft SQL server and analyses were completed in STATA V16.

## Results

### Study patients

There were 10 932 patients identified as undergoing nephrostomy with a concurrent diagnosis of metastatic cancer. Median age was 70 years old (IQR 60–78). The majority were male (58%) (table 1). 57.7% of patients had no recorded comorbidities. Patients predominately had genitourinary cancers (53.2%), followed by gynaecological cancers (18.9%).

**Table 1:** Demographics details of the study patients

Demographic category		Patients	Percentage
Sex	Male	6341	58.0
	Female	4591	42.0
Age (years)	18–29	102	0.9
	30–39	294	2.7
	40–49	658	6.0
	50–59	1475	13.5
	60–69	2831	25.9
	70+	5572	51.0
Ethnicity	White	9954	91.1
	Asian	328	3.0
	Other minority ethnicity	370	3.4
	Unknown	280	2.6
Comorbidity score	0	6307	57.7
	1–4	1379	12.6
	5+	3246	29.7
IMD deprivation quintiles	1 (most deprived)	2059	18.8
	2	2142	19.6
	3	2221	20.3
	4	2336	21.4
	5 (least deprived)	2174	19.9
Procedure year	2010–2011	2124	19.4
	2012–2013	2224	20.3
	2014–2015	2186	20.0
	2016–2017	2173	19.9
	2018–2019	2225	20.4
	Medium	2927	26.8
	High	6781	62.0
Inpatient/outpatient	Inpatient	9936	90.9
	Outpatient	996	9.1
Cancer type	Genitourinary	5812	53.2
	Gynaecological oncology	2061	18.9
	Digestive	1767	16.2
	Other	1292	11.8
Provider cancer volume tertile	1–1576	1694	15.5
	1577–3079	3223	29.5
	3080+	6015	55.0
Specialty	Urology	5106	46.7
	Medicine	2423	22.2
	Oncology	1692	15.5
	General surgery	885	8.1
	Gynaecology/gynaecological oncology	345	3.2
	Other	481	4.4
Complications	Bleeding	885	8.1
	Infection	729	6.7
	Exchange/reinsertion of nephrostomy	1365	12.5
Nephrostomy performed at provider with radiotherapy unit		5511	50.4
Mortality 14 days		717	6.6
Mortality 30 days		1701	15.6
Treatment received	Chemotherapy and surgery	1617	14.8
	Chemotherapy only	1157	10.6
	Surgery only	3172	29.0
	No chemotherapy or surgery	4986	45.6
Total patients		10 932	

IMDIndex of Multiple Deprivation

Outpatient or daycase nephrostomy was performed in only 9.1% of the cohort; of those with an inpatient nephrostomy, 78.7% were during an unplanned or emergency admission and median time to nephrostomy for such patients was 3 days (IQR 1–7).

### Mortality

**Figure 2:**
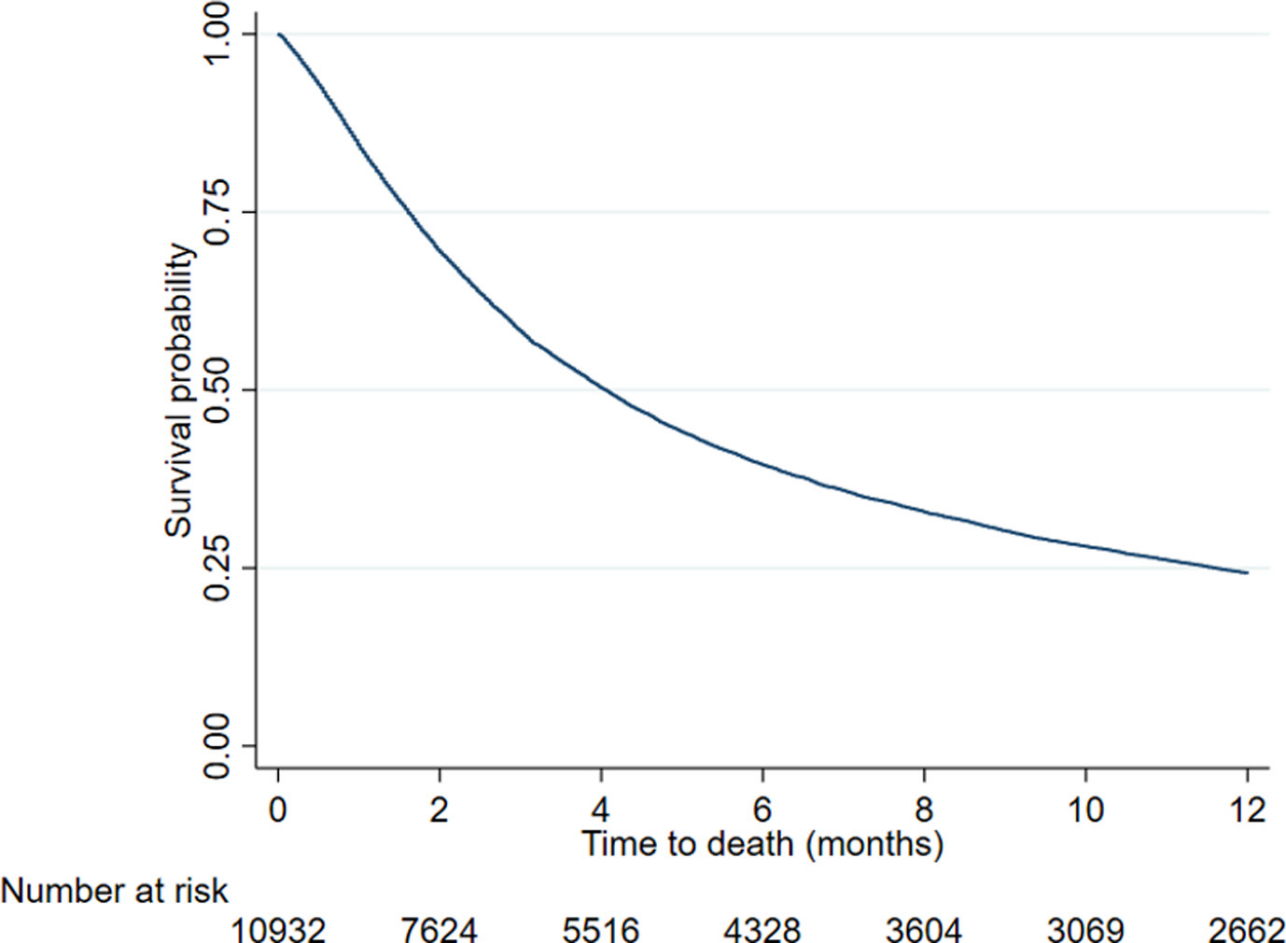
Unadjusted Kaplan-Meir survival curve for time to death (months) following insertion of nephrostomy.

**Figure 3:**
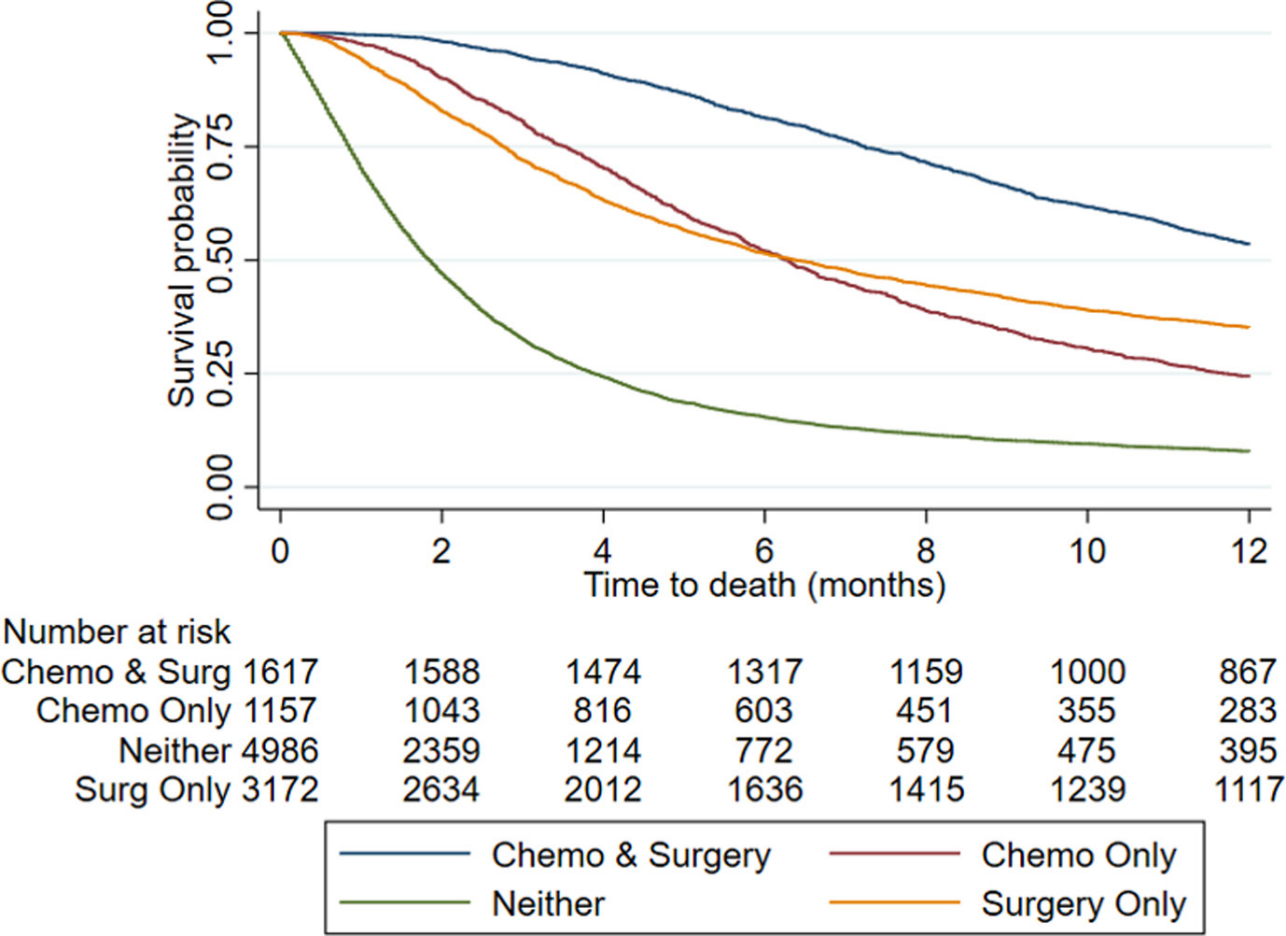
Unadjusted Kaplan-Meir survival curve for time to death (months) following insertion of nephrostomy with cohort split by further treatment received.

Median survival was 124 (IQR 49–354 days (figure 2). Median survival for patients who have chemotherapy or surgery is 293 days (155 571) and 274 days (113 672), respectively (figure 3), compared with 56 days (26 118) for patients who have no treatment. Crude mortality data are described in table 2.

**Table 2:** Descriptive data for deaths following nephrostomy

		N	Death within 14 days	In hospital death within 14 days	Death within 30 days	In hospital death within 30 days
Total		10 932	717 (6.6%)	587 (5.4%)	1701 (15.6%)	1123 (10.3%)
Inpatient nephrostomy(n=9936)	Elective inpatient	2120	85 (4.0%)	578 (3.6%)	205 (9.7%)	146 (6.9%)
	Emergency inpatient	7816	620 (7.9%)	502 (6.4%)	1451 (18.6%)	956 (12.2%)
Outpatient nephrostomy		996	12 (1.2%)	9 (0.9%)	45 (4.5%)	21 (2.1%)

A 30-day mortality was associated with the following variables: inpatient nephrostomy (OR 3.76, 95% CI 2.75 to 5.14, p<0.001); increasing comorbidity (score 1–4 (OR 1.27, 95% CI 1.08 to 1.50, p=0.003), score 5+ (OR 1.29, 95% CI 1.14 to 1.45), p<0.001)) and under the care of specialties other than urology (medicine (OR 2.10, 95% CI 1.84 to 2.40, p<0.001), oncology (OR 1.80, 95% CI 1.51 to 2.15, p<0.001), general surgery (OR 1.62, 95% CI 1.32 to 1.98, p<0.001) and gynaecology/gynaeoncology (OR 1.66, 95% CI 1.21 to 2.28, p=0.002) and older age compared with patients over 70 (age 18–29 (OR 0.36, 95% CI 0.16 to 0.79, p=0.010), age 30–39 (OR 0.68, 95% CI 0.47 to 0.99, p=0.043), age 40–49 (OR 0.65, 95% CI 0.51 to 0.85, p=0.001)) (table 3).

**Table 3:** Multivariable logistic regression analysis of factors associated with death within 30 days of nephrostomy

Demographic category		OR	P value	95% CI
Sex	Male (baseline)			
	Female	1.03	0.718	0.89 to 1.19
Age (years)	18–29	0.36	**0.010**	0.16 to 0.79
	30–39	0.68	**0.043**	0.47 to 0.99
	40–49	0.65	**0.001**	0.51 to 0.85
	50–59	0.85	0.068	0.72 to 1.01
	60–69	0.91	0.160	0.80 to 1.04
	70+ (baseline)			
Ethnicity	White (baseline)			
	Asian	0.73	0.066	0.52 to 1.02
	Other minority ethnicity	0.78	0.124	0.57 to 1.07
	Unknown	1.64	**0.001**	1.23 to 2.21
Comorbidity score	0 (baseline)			
	1–4	1.27	**0.003**	1.08 to 1.50
	5+	1.29	**<0.001**	1.14 to 1.45
IMD deprivation quintiles	1 (most deprived) (baseline)			
	2	1.20	**0.036**	1.01 to 1.43
	3	1.20	**0.037**	1.01 to 1.42
	4	1.05	0.615	0.88 to 1.24
	5 (least deprived)	1.04	0.695	0.87 to 1.24
Procedure year	2010–2011 (baseline)			
	2012–2013	1.17	0.058	0.99 to 1.38
	2014–2015	0.98	0.831	0.83 to 1.16
	2016–2017	0.93	0.379	0.78 to 1.10
	2018–2019	0.89	0.167	0.75 to 1.05
Provider cancer volume tertile	1–1576 (baseline)			
	1577–3079	1.00	0.957	0.85 to 1.18
	3080+	0.87	0.106	0.73 to 1.03
Inpatient/outpatient	Inpatient	3.76	**<0.001**	2.75 to 5.14
	Outpatient (baseline)			
Cancer type	Genitourinary (baseline)			
	Gynaecological oncology	1.12	0.252	0.92 to 1.37
	Digestive	0.99	0.887	0.84 to 1.17
	Other	1.75	**<0.001**	1.47 to 2.09
Specialty	Urology (baseline)			
	Medicine	2.10	**<0.001**	1.84 to 2.40
	Oncology	1.80	**<0.001**	1.51 to 2.15
	General surgery	1.62	**<0.001**	1.32 to 1.98
	Gynaecology/gynaecological oncology	1.66	**0.002**	1.21 to 2.28
	Other	1.72	**<0.001**	1.29 to 2.30
Nephrostomy performed at provider with radiotherapy unit		0.90	0.134	0.79 to 1.03

* p values < 0.001 are in bold.

IMDIndex of Multiple Deprivation

### Chemotherapy

4986 (45.6%) patients had neither surgery nor chemotherapy following nephrostomy. 25.4% of patients received chemotherapy of any type within 6 months of index nephrostomy: 44.7% of outpatient nephrostomy patients but only 23.4% of inpatient nephrostomy patients.

Factors associated with later receiving chemotherapy (table 4) included: younger age(age 18–29 (OR 4.04, 95% CI 2.66 to 6.12, p<0.001), age 30–39 (OR 3.07, 95% CI 2.37 to 3.97, p<0.001), age 40–49 (OR 2.80, 95% CI 2.33 to 3.38, p<0.001), age 50–59 (OR 2.49, 95% CI 2.16 to 2.86, p<0.001), age 60–69 (OR 2.12, 95% CI 1.89 to 2.38, p<0.001)); less deprivation (IMD deprivation quintile 4 (OR 1.29, 95% CI 1.11 to 1.50, p=0.001), IMD deprivation quintile 5 (OR 1.58, 95% CI 1.36 to 1.84, p<0.001)); nephrostomy in a more recently (years 2016/2017 (OR 1.31, 95% CI 1.13 to 1.52, p=0.001), years 2018/2019 (OR 1.44, 95% CI 1.24 to 1.67, p<0.001)); gynaecological cancer (OR 1.25, 95% CI 1.06 to 1.47, p=0.009); under the care of oncology (OR 1.60, 95% CI 1.40 to 1.83, p<0.001) or gynaeoncology (OR 1.64, 95% CI 1.28 to 2.10, p<0.001), rather than medicine (OR 0.63, 95% CI 0.55 to 0.72, p<0.001); increasing comorbidity (Charlson score 1–4 (OR 0.79, 95% CI 0.68 to 0.91, p=0.001), Charlson score 5 (OR 0.53, 95% CI 0.47 to 0.59, p<0.001)) and inpatient nephrostomy (OR 0.41, 95% CI 0.35 to 0.48, p<0.001).

**Table 4:** Multivariable logistic regression for analysis of factors associated with chemotherapy following nephrostomy

Demographic category		OR	P value	95% CI
Sex	Male (baseline)			
	Female	1.09	0.176	0.96 to 1.24
Age (years)	18–29	4.04	**<0.001**	2.66 to 6.12
	30–39	3.07	**<0.001**	2.37 to 3.97
	40–49	2.80	**<0.001**	2.33 to 3.38
	50–59	2.49	**<0.001**	2.16 to 2.86
	60–69	2.12	**<0.001**	1.89 to 2.38
	70+ (baseline)			
Ethnicity	White (baseline)			
	Asian	0.94	0.645	0.72 to 1.23
	Other minority ethnicity	1.04	0.734	0.81 to 1.35
	Unknown	0.63	**0.004**	0.46 to 0.86
Comorbidity score	0 (baseline)			
	1–4	0.79	**0.001**	0.69 to 0.91
	5+	0.53	**<0.001**	0.47 to 0.59
IMD deprivation quintiles	1 (most deprived) (baseline)			
	2	1.18	**0.034**	1.01 to 1.37
	3	1.07	0.386	0.92 to 1.25
	4	1.29	**0.001**	1.11 to 1.50
	5 (least deprived)	1.58	**<0.001**	1.36 to 1.84
Procedure year	2010–2011 (baseline)			
	2012–2013	1.06	0.441	0.91 to 1.23
	2014–2015	1.14	0.086	0.98 to 1.32
	2016–2017	1.31	**<0.001**	1.13 to 1.52
	2018–2019	1.44	**<0.001**	1.24 to 1.67
Provider cancer volume tertile	1–1576 (baseline)			
	1577–3079	0.94	0.471	0.81 to 1.10
	3080+	1.07	0.428	0.91 to 1.25
Inpatient/outpatient	Inpatient	0.41	**<0.001**	0.35 to 0.48
	Outpatient (baseline)			
Cancer type	Genitourinary (baseline)			
	Gynaecological oncology	1.25	**0.009**	1.06 to 1.47
	Digestive	1.08	0.264	0.94 to 1.24
	Other	0.71	**<0.001**	0.59 to 0.84
Specialty	Urology (baseline)			
	Medicine	0.63	**<0.001**	0.55 to 0.72
	Oncology	1.60	**<0.001**	1.40 to 1.83
	General surgery	0.85	0.085	0.71 to 1.02
	Gynaecology/gynaecological oncology	1.64	**<0.001**	1.28 to 2.10
	Other	0.82	0.092	0.65 to 1.03
Nephrostomy performed at provider with radiotherapy unit		1.08	0.196	0.96 to 1.21

p values < 0.001 are in bold.

IMDIndex of Multiple Deprivation

### Surgery

4789 (43.8%) of patients had later abdominopelvic surgery. Factors associated with proceeding to surgery were (table 5): younger age compared with those over 70 (age 18–29 (OR 1.51, 95% CI 1.01 to 2.27, p=0.045), age 30–39 (OR 1.51, 95% CI 1.18 to 1.94, p=0.001), age 40–49 (OR 1.47, 95% CI 1.23 to 1.74, p<0.001), age 50–59 (OR 1.34, 95% CI 1.19 to 1.52, p<0.001), age 60–69 (OR 1.20, 95% CI 1.09 to 1.32, p<0.001)); female ((OR 1.13, 95% CI 1.01 to 1.26, p=0.029)); high comorbidity score, score 5+ (OR 0.79, 95% CI 0.72 to 0.86), p<0.001)); inpatient nephrostomy (OR 0.82, 95% CI 0.72 to 0.95, p=0.007); gynaeoncology cancer (OR 0.86, 95% CI 0.74 to 0.99, p=0.037), other cancers (OR 0.75, 95% CI 0.65 to 0.86, p<0.001)) and under the care of medicine (OR 0.69, 95% CI 0.63 to 0.77, p<0.001) or oncology (OR 0.58, 95% CI 0.51 to 0.66, p<0.001).

**Table 5:** Multivariable logistic regression analysis of factors associated with surgery following nephrostomy

Demographic category		OR	P value	95% CI
Sex	Male (Baseline)			
	Female	1.13	**0.029**	1.01 to 1.26
Age (years)	18–29	1.51	**0.045**	1.01 to 2.27
	30–39	1.52	**0.001**	1.18 to 1.94
	40–49	1.47	**<0.001**	1.23 to 1.74
	50–59	1.34	**<0.001**	1.19 to 1.52
	60–69	1.20	**<0.001**	1.09 to 1.32
	70+ (Baseline)			
Ethnicity	White (Baseline)			
	Asian	1.21	0.102	0.96 to 1.52
	Other minority ethnicity	1.15	0.197	0.93 to 1.43
	Unknown	0.76	**0.036**	0.59 to 0.98
Comorbidity score	0 (Baseline)			
	1–4	0.89	0.056	0.79 to 1.00
	5+	0.79	**<0.001**	0.72 to 0.86
IMD deprivation quintiles	1 (Most Deprived) (Baseline)			
	2	1.00	0.997	0.88 to 1.13
	3	1.09	0.178	0.96 to 1.24
	4	1.10	0.135	0.97 to1.25
	5 (Least Deprived)	1.04	0.542	0.92 to 1.18
Procedure year	2010–11 (Baseline)			
	2012–13	0.93	0.262	0.83 to 1.05
	2014–15	0.86	**0.017**	0.76 to 0.97
	2016–17	0.86	**0.013**	0.76 to 0.97
	2018–19	0.43	**<0.001**	0.38 to 0.49
Provider cancer volume tertile	1–1576 (Baseline)			
	1577–3079	0.91	0.158	0.81 to 1.04
	3080+	1.02	0.740	0.90 to 1.16
Inpatient/Outpatient	Inpatient	0.82	**0.007**	0.72 to 0.95
	Outpatient (Baseline)			
Cancer type	Genitourinary (Baseline)			
	Gynaecological oncology	0.86	**0.037**	0.74 to 0.99
	Lower GI	0.94	0.350	0.84 to 1.06
	Other	0.75	**<0.001**	0.65 to 0.86
Specialty	Urology (Baseline)			
	Medicine	0.69	**<0.001**	0.63 to 0.77
	Oncology	0.58	**<0.001**	0.51 to 0.66
	General Surgery	0.93	0.320	0.80 to 1.08
	Gynaecology/gynaecological oncology	1.01	0.920	0.80 to 1.29
	Other	0.75	**0.005**	0.61 to 0.92
Nephrostomy performed at provider with radiotherapy unit		1.06	0.248	0.96 to 1.16

p values < 0.001 are in bold.

IMDIndex of Multiple Deprivation

### Complications of nephrostomy

2650 (24.2%) patients had at least one direct complication of nephrostomy. The most common complication was an early exchange of nephrostomy in 1365 (12.5%) patients, followed by bleeding in 885 (8.1%) and pyelonephritis in 729 (6.7%). In patients with an early exchange of nephrostomy, the median time to exchange was 10 days (IQR 5–25).

## Discussion

This study has identified high mortality within 30 days of nephrostomy in the context of MUO in metastatic malignancy, particularly in patients during an emergency admission; with the majority of such patients dying as an inpatient. This study did not include locally advanced non-metastatic cancers, wherein a significant proportion of patients will have renal decompression. Survival for patients with MUO has been estimated to be between 3 and 7 months following diagnosis.^5^ This study identified a median survival of 4 months, indicating that insertion of a nephrostomy in this particular cohort of patients does not necessarily prolong life; this poor survival is in spite of improving biochemical renal function.^6^ Survival was markedly lower in patients not going on to have further treatment with chemotherapy or surgery.

Less than half of patients with MUO have an increased quality of life as a result of nephrostomy.^6^ Furthermore, if a nephrostomy is sited, it is estimated that patients spend 20% of their remaining lifetime as an inpatient in hospital^7^; with patients spending 14 days in hospital just for the nephrostomy itself.^8^ The majority of patients, despite nephrostomy or another modality of urinary diversion, have persisting symptoms from their cancer.^9^ Therefore, the insertion of percutaneous nephrostomy appears to have limited positive impact on length and quality of life, without commensurate symptom relief for this cohort of patients.

Complications from percutaneous urinary diversion should not be underestimated. Almost one in four patients in this study had a direct complication of nephrostomy insertion; delayed complications were not captured in this study and may reveal a higher rate. While most adverse effects pertain to displacement or blockage of drainage tubes, severe infection and bleeding can occur; almost half of patients may experience at least a single episode of pyelonephritis.^10^ Migration and extrusion of nephrostomy tubes can sometimes impact on the complex care needs of patients with external urinary drainage and emphasises the importance of fixation of the nephrostomy tubes to the skin. Furthermore, only 1 in 10 patients had a chance of internalisation of renal drainage, with an antegrade stent, further burdening patients and community care services.

Given that there is no extended survival or symptomatic improvement, nephrostomy is only indicated in circumstances to facilitate effective treatment, radical or palliative. A large proportion of patients with MUO receive no additional therapy beyond renal decompression.^11 12^

There have been a number of studies that have aimed to develop a prognostic model for predicting poor outcomes following nephrostomy for patients with ureteric obstruction secondary to malignant disease,^13^,^17^ considering both clinical presentations as well as biochemical parameters. While there is some overlap between factors in the studies identified, there is no consistently examined group of factors; hence there is no single approved risk prediction strategy in place at present. This highlights the complexity of decision-making for such patients and the importance of multidisciplinary cancer team input with individualised care plans for all such patients. We believe there is an urgent need for comprehensive prospective real-world data to better evaluate this clinical challenge.

Patients receiving nephrostomy as an inpatient, particularly in emergency settings, have a higher likelihood of dying within 30 days and are less likely to receive subsequent surgery or chemotherapy. Nephrostomy is performed by interventional radiology and requested by a wide variety of inpatient teams. Therefore, inpatient teams with limited experience of urinary diversion and complex oncological disease may be requesting nephrostomy insertion. It should be noted that only 1 in 10 patients had outpatient nephrostomy, yet half of these patients went on to receive chemotherapy. This is indicative of the improved outcomes in patients with planned procedures. Given that this study has identified that patients wait several days for nephrostomy in emergency settings; there is time for these patients to be discussed in a multidisciplinary cancer team meeting setting or in the context of a best interests meeting, to prevent patients undergoing futile procedures.

The volume of procedures at provider level can impact the outcomes of procedures in patients with poor prognosis cancers. For example, Endoscopic retrograde cholangio-pancreatograpy (ERCP) performed in the context of un-resectable cancer was associated with 30-day mortality in low volume providers,^18^ but no volume effect was seen in this study. The volume of cancer patients treated by a provider was used as a surrogate for experience in nephrostomy decision-making. Patients are regularly referred between hospital providers for nephrostomy insertion in the UK as interventional radiology services may be limited outside of tertiary care settings, particularly outside of normal working hours. No volume effect was observed in this study; this may be due to the fact that in HES we cannot be entirely sure which centre took the decision to site a nephrostomy.

Adequate counselling of patients, and by extension, consent prior to any intervention is paramount, underscored by the Montgomery ruling.^19^ Not only should risks, with the inclusion of impaired quality of life, be explained in detail, but comprehensive discussions must include adequate information pertaining to alternatives. This should not discount ‘doing nothing’ as an option, given the relatively short postnephrostomy life expectancy we have demonstrated. Individual disciplines managing patients with MUO in the acute setting are not necessarily equipped with the knowledge or skillset to manage this complex scenario. Despite several descriptions, heterogeneous cohorts and retrospective analyses mean that the existing data does not adequately inform discussions.^20^ This is also compounded by differing approaches that urologists and oncologists are likely to take^21^ and the potential for patients (and family) to be overwhelmed to the extent that it impairs decision-making.^22^ This is a complex process in a vulnerable patient group, where the default of simply decompressing the ‘obstructed urinary tract’ is no longer acceptable and a ‘realistic medicine’^23^ approach should be considered.

The main strengths of this study are that this was a nationwide sample of all patients undergoing nephrostomy for MUO and it is very unlikely that such procedures would be undertaken in the independent sector outside the NHS, and therefore, nearly all cases were captured. This makes the cohort characteristic of a real-world population. Further to this, the power of the study is high, as indicated by the narrow CIs and inferences can be made with more confidence. Importantly, data, in particular routine administrative data, in England is of extremely high quality. HES data pertaining to such procedures have been validated previously.^24^ However, there are a number of limitations to this study. As HES data are primarily administrative, there are no biochemical or histopathological data available, preventing a clear understanding of indication for nephrostomy. Furthermore, patients cannot be identified, therefore, qualitative research regarding quality of life cannot be undertaken. Multidisciplinary cancer team outcome data cannot be reviewed to examine the predicted prognosis or whether the patient was already considered for palliative care at time of nephrostomy; additionally, whether the decision was ratified by the multidisciplinary team. Although this study provides a picture of outcomes for this cohort of patients, it is not possible to control for all potential confounding variables.

## Conclusions

Large numbers of patients undergo nephrostomy with no further treatment, with a significant proportion experiencing death within a short time frame and a high complication rate. The decision to perform nephrostomy in such patients is a complex one and should be reviewed in a multidisciplinary cancer team setting, with surgical, oncological and palliative care input. In acute inpatient settings, it should be discussed with expert teams, should a multidisciplinary cancer team meeting not be available within the required timeframe. Care must be taken to adequately counsel and inform patients of the advantages of nephrostomy in the context of such a poor prognosis.

## supplementary material

10.1136/spcare-2024-004937 online supplemental file 1

## Data Availability

All data relevant to the study are included in the article.
