# Supplementary material for: High early mortality following percutaneous nephrostomy in metastatic cancer: a national analysis of outcomes
Source: BMJ Support Palliat Care. 2024 Jul 13;14(e2):e004937. doi: 10.1136/spcare-2024-004937 (PMC11672021; doi:10.1136/spcare-2024-004937)
Supplement: online supplemental file 1 [file spcare-14-e2-s001.doc]

STROBE Statement—Checklist of items that should be included in reports of ***cohort studies***

|  |  | Item No | Recommendation |
| --- | --- | --- | --- |
| ✔ | **Title and abstract** | 1 | (*a*) Indicate the study’s design with a commonly used term in the title or the abstract |
| ✔ |  |  | (*b*) Provide in the abstract an informative and balanced summary of what was done and what was found |
|  | Introduction | | |
| ✔ | Background/rationale | 2 | Explain the scientific background and rationale for the investigation being reported |
| ✔ | Objectives | 3 | State specific objectives, including any prespecified hypotheses |
|  | Methods | | |
| ✔ | Study design | 4 | Present key elements of study design early in the paper |
| ✔ | Setting | 5 | Describe the setting, locations, and relevant dates, including periods of recruitment, exposure, follow-up, and data collection |
| ✔ | Participants | 6 | (*a*) Give the eligibility criteria, and the sources and methods of selection of participants. Describe methods of follow-up |
| n/a |  |  | (*b*) For matched studies, give matching criteria and number of exposed and unexposed |
| ✔ | Variables | 7 | Clearly define all outcomes, exposures, predictors, potential confounders, and effect modifiers. Give diagnostic criteria, if applicable |
| ✔ | Data sources/ measurement | 8* | For each variable of interest, give sources of data and details of methods of assessment (measurement). Describe comparability of assessment methods if there is more than one group |
| ✔ | Bias | 9 | Describe any efforts to address potential sources of bias |
| ✔ | Study size | 10 | Explain how the study size was arrived at |
| ✔ | Quantitative variables | 11 | Explain how quantitative variables were handled in the analyses. If applicable, describe which groupings were chosen and why |
| ✔ | Statistical methods | 12 | (*a*) Describe all statistical methods, including those used to control for confounding |
|  |  |  | (*b*) Describe any methods used to examine subgroups and interactions |
|  |  |  | (*c*) Explain how missing data were addressed |
|  |  |  | (*d*) If applicable, explain how loss to follow-up was addressed |
|  |  |  | (*e*) Describe any sensitivity analyses |
|  | Results | | |
| ✔ | Participants | 13* | (a) Report numbers of individuals at each stage of study—eg numbers potentially eligible, examined for eligibility, confirmed eligible, included in the study, completing follow-up, and analysed |
|  |  |  | (b) Give reasons for non-participation at each stage |
|  |  |  | (c) Consider use of a flow diagram |
| ✔ | Descriptive data | 14* | (a) Give characteristics of study participants (eg demographic, clinical, social) and information on exposures and potential confounders |
|  |  |  | (b) Indicate number of participants with missing data for each variable of interest |
|  |  |  | (c) Summarise follow-up time (eg, average and total amount) |
| ✔ | Outcome data | 15* | Report numbers of outcome events or summary measures over time |
| ✔ | Main results | 16 | (*a*) Give unadjusted estimates and, if applicable, confounder-adjusted estimates and their precision (eg, 95% confidence interval). Make clear which confounders were adjusted for and why they were included |
|  |  |  | (*b*) Report category boundaries when continuous variables were categorized |
|  |  |  | (*c*) If relevant, consider translating estimates of relative risk into absolute risk for a meaningful time period |
| ✔ | Other analyses | 17 | Report other analyses done—eg analyses of subgroups and interactions, and sensitivity analyses |
|  | Discussion | | |
| ✔ | Key results | 18 | Summarise key results with reference to study objectives |
| ✔ | Limitations | 19 | Discuss limitations of the study, taking into account sources of potential bias or imprecision. Discuss both direction and magnitude of any potential bias |
| ✔ | Interpretation | 20 | Give a cautious overall interpretation of results considering objectives, limitations, multiplicity of analyses, results from similar studies, and other relevant evidence |
| ✔ | Generalisability | 21 | Discuss the generalisability (external validity) of the study results |
|  | Other information | | |
| ✔ | Funding | 22 | Give the source of funding and the role of the funders for the present study and, if applicable, for the original study on which the present article is based |

*Give information separately for exposed and unexposed groups.
